# Preoperative handgrip strength can predict early postoperative shoulder function in patients undergoing arthroscopic rotator cuff repair

**DOI:** 10.1186/s13018-024-04750-8

**Published:** 2024-04-30

**Authors:** Yu-Cheng Liu, Shu-Wei Huang, Christopher R. Adams, Chung-Ying Lin, Yu-Pin Chen, Yi-Jie Kuo, Tai-Yuan Chuang

**Affiliations:** 1 Department of General Medicine, Shuang Ho Hospital, Taipei Medical University, New Taipei City, Taiwan; 2 Department of Applied Science, National Taitung University, Taitung City, Taitung County Taiwan; 3 Arthrex, Inc., Naples, FL USA; 4 Orthopaedic Department, Naples Community Hospital, Naples, FL USA; 5 Institute of Allied Health Sciences, College of Medicine, National Cheng Kung University, Tainan, Taiwan; 6 Department of Occupational Therapy, College of Medicine, National Cheng Kung University, Tainan, Taiwan; 7 Department of Public Health, College of Medicine, National Cheng Kung University Hospital, National Cheng Kung University, Tainan, Taiwan; 8 Department of Orthopedics, Taipei Municipal Wan Fang Hospital, Taipei Medical University, Taipei, Taiwan; 9 Department of Orthopedics, School of Medicine, College of Medicine, Taipei Medical University, Taipei, Taiwan

**Keywords:** Rotator cuff tear, Arthroscopic rotator cuff repair, Handgrip strength, Postoperative outcome, Shoulder functional outcome, Postoperative shoulder function

## Abstract

**Background:**

Rotator cuff tears (RCTs) are a common musculoskeletal disorder, and arthroscopic rotator cuff repair (ARCR) is widely performed for tendon repair. Handgrip strength correlates with rotator cuff function; however, whether preoperative grip strength can predict functional outcomes in patients undergoing ARCR remains unknown. This study aimed to investigate the correlation between preoperative grip strength and postoperative shoulder function following ARCR.

**Methods:**

A total of 52 patients with full-thickness repairable RCTs were prospectively enrolled. Baseline parameters, namely patient characteristics and intraoperative findings, were included for analysis. Postoperative shoulder functional outcomes were assessed using the Quick Disabilities of the Arm, Shoulder, and Hand (QDASH) questionnaire and Constant–Murley scores (CMSs). Patients were followed up and evaluated at three and six months after ARCR. The effects of baseline parameters on postoperative outcomes were measured using generalized estimating equations.

**Results:**

At three and six months postoperatively, all clinical outcomes evaluated exhibited significant improvement from baseline following ARCR. Within 6 months postoperatively, higher preoperative grip strength was significantly correlated with higher CMSs (β = 0.470, *p* = 0.022), whereas increased numbers of total suture anchors were significantly correlated with decreased CMSs (β =  − 4.361, *p* = 0.03). Higher body mass index was significantly correlated with higher postoperative QDASH scores (β = 1.561, *p* = 0.03) during follow-up.

**Conclusions:**

Higher baseline grip strength predicts more favorable postoperative shoulder function following ARCR. A preoperative grip strength test in orthopedic clinics may serve as a predictor for postoperative shoulder functional recovery in patients undergoing ARCR.

## Background

Rotator cuff tears (RCTs) are one of the most common musculoskeletal disorders affecting shoulder joint in orthopedic clinics. The prevalence of RCTs is approximately 20% in the general population and increases with age [1–3]. Shoulder pain due to RCTs can strongly affect shoulder function and restrict daily activities, which is associated with lower health-related quality of life and higher health-care costs [4, 5]. Surgical interventions of RCT involve open tendon repair, mini-open tendon repair, and arthroscopic rotator cuff repair (ARCR) [6]; ARCR is currently the most widely performed surgical procedure for RCT, and the postoperative outcomes are generally favorable [7]. The majority of patients undergoing ARCR experience pain relief and substantial improvements in shoulder function and quality of life after surgical intervention [7].

Despite the generally positive outcomes, the postoperative recovery and rehabilitation process after ARCR can be lengthy [8, 9], and numerous factors have been reported to affect patients’ functional recovery and overall outcomes following ARCR. Identifying prognostic factors for postoperative outcomes is challenging but essential for appropriate clinical decision-making before surgery, considering recovery from pain and restoration of function may not go hand in hand with the appearance of the affected tendon at imaging following tendon repair [10, 11]. In addition, identifying prognostic factors can also help patients understand what to expect during the recovery period, thus improving doctor–patient communication and patient satisfaction. In the literature, the potential predictors of postoperative functional recovery and outcomes following ARCR include age, comorbidities, obesity, preoperative rotator cuff function and strength, workers’ compensation claims, and tear size [12–16].

Handgrip strength is the maximum force generated by an individual’s hand, which is quantified as the amount of static force applied when the hand squeezes a dynamometer. Studies have identified grip strength as an explanatory and predictive factor of general health outcomes such as generalized strength, disability, and mortality [17, 18]. Grip strength tests have also been conducted in clinical settings to assess functional recovery following stroke and to screen for sarcopenia [19, 20]. In orthopedic research, grip strength predicts surgical and functional outcomes in patients undergoing spine and hip fracture surgery [21–23]. Furthermore, numerous studies have demonstrated the correlation between grip strength and rotator cuff function [24–27]; Manske et al. investigated this correlation in patients with RCT and reported positive correlations between grip strength of the affected side and ipsilateral shoulder abduction and external rotation strength [27].

Although grip strength is correlated with rotator cuff function, no current study has investigated whether baseline grip strength can predict postoperative outcomes in patients undergoing arthroscopic repair for RCT. Therefore, the primary aim of this study is to investigate the correlation between preoperative grip strength and postoperative shoulder function in patients undergoing ARCR. We hypothesized that a higher baseline grip strength would predict more favorable shoulder function following ARCR.

## Methods

### Study design

This prospective study recruited patients who underwent ARCR at a single medical center in Taipei, Taiwan, from May 1 to October 31, 2020. Qualified patients were adults aged ≥ 50 years who had ≥ 1-cm full-thickness tears of the supraspinatus tendon that were diagnosed through magnetic resonance imaging and who received ARCR. Patients were excluded if they (1) received open repair of RCT, (2) had tears of the supraspinatus tendon that were shorter than 1 cm, or (3) had irreparable massive RCT confirmed during operation. This study was conducted in accordance with the code of ethics of the World Medical Association (Declaration of Helsinki) and was approved by the Ethics Committee of Taipei Medical University (TMU-JIRB N202002025). All participants provided written consent to participate in this study and to have their data published.

Demographic data, namely age, sex, body mass index (BMI), symptom duration, and underlying comorbidities, which were represented using Charlson comorbidity index (CCI) values, were collected at baseline before the operation [28]. While the patient was sitting in bed or on a chair with the elbow flexed and the wrist in a neutral position, preoperative maximum handgrip strength was measured for each patient by using a Jamar Hydraulic Dynamometer (Sammons Preston, USA). Patients were instructed to grip the device as hard as possible three times by using each hand [29]; the best measurement of the affected side was included in the analysis. Surgical findings, namely the size of the supraspinatus tendon tear, concomitant tears of the subscapularis tendon, and overall number of suture anchors for tendon repair, were also included in the analysis.

The primary outcome of this study was the correlation of preoperative grip strength with clinical outcome measures. The secondary outcomes were the correlations of other variables, namely baseline demographic data and surgical findings, with outcome measures.

### Outcome assessment

All clinical outcomes, namely patient-reported pain, quality of life, and shoulder function, were measured for each patient at baseline and at three and six months postoperatively by an independent investigator blinded to the patients’ diagnosis and surgical findings. Pain was measured using a visual analog scale (VAS), which quantifies pain severity from 0 (*no pain*) to 10 (*worst pain*) [30]. Quality of life was evaluated using the EuroQol-5D (EQ-5D) questionnaire [31]; a higher EQ-5D score indicates higher quality of life. Shoulder function was measured using the patient-reported Quick Disabilities of the Arm, Shoulder, and Hand (QDASH) questionnaire and the Constant–Murley score (CMS). The QDASH consists of 11 items used to assess the physical function and symptoms of patients with upper limb disorders [32]. The CMS, which is used to determine functionality after the treatment of a shoulder injury, is a 100-point scale consisting of four subscales: pain, activities of daily living, strength, and range of motion [33]. Lower QDASH scores and higher CMSs indicate more favorable shoulder function.

### Operative techniques

All patients underwent ARCR performed by one experienced orthopedic shoulder specialist who had performed arthroscopic surgery for more than 3,000 patients with RCTs. All procedures were performed in the lateral decubitus position under axillary and subscapular nerve blocks. A standard posterior viewing portal and anterior portal were created for thorough arthroscopic examination and assessment of intra-articular pathology involving the coracoid process, middle glenohumeral ligament, and supraspinatus, infraspinatus, and subscapularis tendons. Rotator cuff tendon repair was performed with a single-row technique in all patients.

#### Subscapularis tendon repair

An anterosuperolateral portal, serving as the primary working portal, was created at a 5° to 10° angle of approach toward the lesser tuberosity bone bed. The extent of the subscapularis tear was intraoperatively determined through direct arthroscopic visualization after debridement of the degenerated tendon edges. A bone bed for subscapularis tendon repair on the lesser tuberosity was prepared. A suture anchor was placed at the anterolateral aspect of the bone footprint on the lesser tuberosity. The retrograde suture passer was used to pass the suture. The sutures for each anchor were tied as they were placed.

#### Subacromial decompression

An arthroscope was inserted into the subacromial space through the posterior portal. To alleviate bursitis, thorough bursectomy was performed using a shaver. Subacromial decompression was performed using a burr through a lateral portal. A flat undersurface to the acromion was then created.

#### Supraspinatus and infraspinatus tendon repair

After debridement of the degenerated tendon edges, the extent of the tear was intraoperatively determined through direct arthroscopic visualization. Mediolateral and anteroposterior tension was assessed using a cuff grasper (Arthrex, Naples, FL, USA). To achieve repair that was as tension free as possible, marginal convergence was performed as needed after the tear pattern was determined. The bone bed of the footprint on the greater tuberosity was prepared using a power shaver and burr. The suture anchors were placed on the bone bed after the required number of anchors was determined. The suture was passed 8–12 mm from the cuff margin by using an antegrade suture passer. The sutures for each anchor were tied as they were placed from posterior to anterior.

### Postoperative management and rehabilitation

All patients were provided a sling with a small pillow in the operating room. The sling had to be worn full time for six weeks, except during showers or meals. Forceful elbow and wrist motion were prohibited during daily activities for six weeks. Instructions for home rehabilitation and exercise program were given in the shoulder surgeon’s office. At six weeks postoperatively, the patients could stop using the sling; they began to stretch with forward elevation by performing door sliding and external rotation by using a door. At eight weeks, they began to build strength by performing pushups against a wall. Patients were permitted to use light weights on the basis of their progress. Complete return to unrestricted activities usually occurred at three to six months postoperatively and was based on the initial size of the tear, the strength of the repair, and the patient’s rehabilitation progress.

### Statistical analysis

Sample size calculation was based on following set-ups: (1) type I error at 0.05, (2) power at 0.8, (3) a two-tailed test, and the results of Manske et al. [27]. To reach a power of 0.8, a sample size of 52 was needed. Therefore, we aimed to recruit at least 52 participants at baseline.

Statistical analysis was conducted using SPSS (version 24; IBM, Armonk, NY, USA). Categorical variables are presented as frequencies (percentages), and continuous variables are presented as means ± standard deviations (SDs). A paired *t* test was conducted to compare the repeated measurements of VAS, EQ-5D, QDASH, and CMS at baseline and three and six months postoperatively. All outcomes were analyzed using generalized estimating equations (GEEs) with restricted maximum likelihood estimation, and the analysis was controlled for the effects of time during follow-up. For all tests, two-sided *p* values of < 0.05 were considered statistically significant.

## Results

### Study population selection and patient demographics

**Fig. 1:**
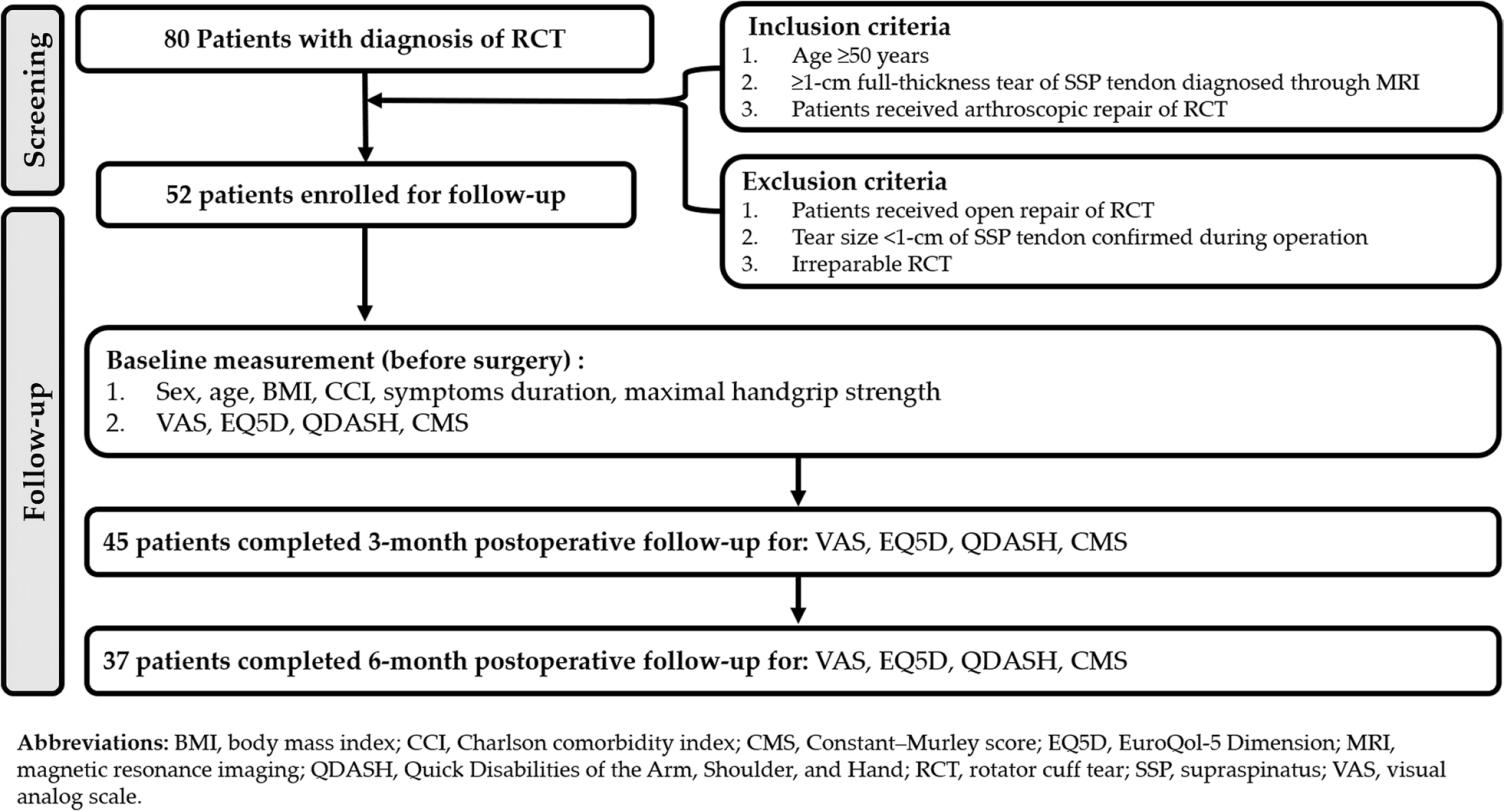
Flowchart of patient selection and follow-up

From May 1 to October 31, 2020, 80 patients were prospectively diagnosed as having RCT and received surgical intervention at a medical center in Taipei, Taiwan. Of these patients, 28 who met the exclusion criteria were excluded; the remaining 52 patients with repairable, full-thickness RCT were included for postoperative follow-up and analysis. Figure 1 presents the flowchart of the selection of the study population. A total of 45 and 37 patients completed the three- and six-month postoperative follow-up, respectively. The exact reason for the patients’ loss-to-follow-up remained unclear. The primary speculated cause was the patients’ perception of symptom amelioration, deeming further follow-up visits unnecessary.

The baseline characteristics of the study population were recorded at baseline before ARCR. The mean age of the patients at the time of surgery was 63.2 years (SD: 7.45). Female patients comprised 75% (n = 39) of the study population. More than half (57.7%; n = 30) of the study population had a symptom duration of more than 12 months before ARCR. The mean baseline handgrip strength was 23.5 kg (SD: 10.1). Surgical findings, namely size of the supraspinatus tendon tear, whether a torn subscapularis tendon was involved, and the number of total suture anchors used for tendon repair, were also recorded. A total of 61.5% (n = 32) of the patients had tears of > 3 cm, and in 42.3% (n = 22) of the patients, a torn subscapularis tendon was involved. The mean number of suture anchors was 2.62 (SD: 1.07). Table 1 presents the patient demographics.

**Table 1:** Patient demographics

Patient demographics* (n = 52)	
Age	63.21 ± 7.45
Sex (women)	39 (75.0%)
Symptom duration	
< 3 months	6 (11.5%)
3–6 months	5 (9.6%)
6–12 months	11 (21.2%)
> 12 months	30 (57.7%)
BMI	24.48 ± 3.30
CCI	1.38 ± 1.27
Baseline handgrip strength	23.50 ± 10.10
Combined SSC tear	22 (42.3%)
SSP tear size	
1–3 cm	20 (38.5%)
> 3 cm	32 (61.5%)
Suture anchor number	2.62 ± 1.07

*BMI* body mass index; *CCI* Charlson comorbidity index; *SSC* subscapularis tendon; *SSP* supraspinatus tendon

*Continuous data are expressed as means ± standard deviations; categorical data are expressed as numbers with percentages

### Outcome measures

**Fig. 2:**
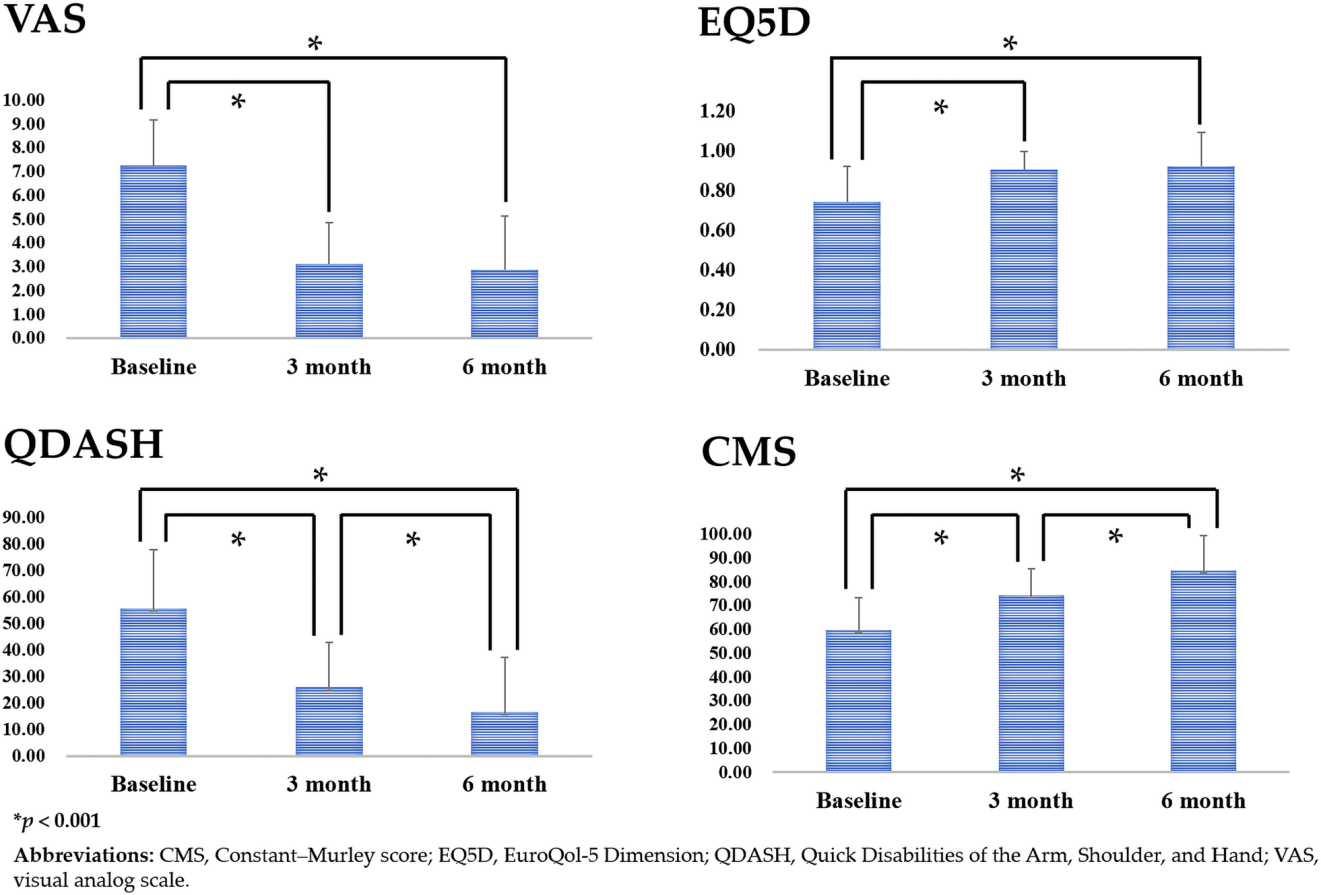
Follow-up outcome scores

The outcome measures were evaluated at baseline and three and six months after ARCR (Fig. 2 and Table 2). Compared with baseline values, all clinical outcomes exhibited significant improvement at three and six months after ARCR (*p* < 0.001; Fig. 2). QDASH scores and CMSs evaluated six months after ARCR exhibited a significant improvement compared with those at three months postoperatively (*p* < 0.001; Fig. 2); however, this improvement was not observed for VAS and EQ5D scores (Fig. 2).

**Table 2:** Outcome scores at baseline and 3 and 6 months after ARCR

Outcome*	Baseline (n = 52)	3 months (n = 45)	6 months (n = 37)
VAS	7.25 ± 1.93	3.13 ± 1.71	2.89 ± 2.23
EQ5D	0.75 ± 0.18	0.91 ± 0.09	0.92 ± 0.17
QDASH	55.86 ± 22.19	25.86 ± 17.14	16.65 ± 20.70
CMS	59.81 ± 13.41	74.53 ± 10.95	84.78 ± 14.80

*ARCR* arthroscopic rotator cuff repair; *CMS* Constant–Murley score; *EQ5D* EuroQol-5 Dimension; *QDASH* Quick Disabilities of the Arm, Shoulder, and Hand; *VAS* visual analog scale

*All outcome scores are expressed as means ± standard deviations

### Correlations of baseline parameters with outcome measures

All the baseline parameters listed in Table 1 were included in the GEE analysis to investigate their relationship with the outcome measures of interest. Table 3 presents the effects of the analyzed baseline parameters on all outcome measures in the six-month postoperative follow-up.

**Table 3:** Correlation of baseline parameters with postoperative outcome scores

Parameter	VAS			EQ5D			QDASH			CMS		
	β	SE	*p*	β	SE	*p*	β	SE	*p*	β	SE	*p*
Age	− 0.052	0.0273	0.056	0.004	0.0023	0.056	− 0.216	0.3026	0.476	0.363	0.1956	0.063
Sex (ref: men)	0.249	0.5576	0.655	− 0.019	0.0393	0.631	11.5	6.7672	0.089	3.182	4.8158	0.509
Duration (ref: < 3 months)												
3–6 month	1.02	0.6592	0.122	− 0.003	0.0439	0.953	− 0.012	6.0597	0.998	0.883	3.4972	0.801
6–12 month	0.921	0.7105	0.195	− 0.033	0.0533	0.541	8.484	8.2792	0.305	− 6.723	4.8353	0.164
> 12 month	0.740	0.8132	0.363	− 0.016	0.0564	0.78	11.222	8.816	0.203	− 7.05	5.8222	0.226
BMI	0.046	0.0562	0.414	− 0.004	0.0036	0.229	**1.561**	**0.7194**	**0.03**	− 0.699	0.4058	0.085
CCI	− 0.189	0.1963	0.335	− 0.01	0.0153	0.515	0.503	2.314	0.828	− 0.485	1.4316	0.735
Baseline handgrip strength	− 0.036	0.0268	0.184	0.001	0.0017	0.718	− 0.06	0.2762	0.828	**0.470**	**0.2052**	**0.022**
Combined SSC tear (ref: no)	0.348	0.3839	0.364	− 0.002	0.0318	0.961	− 1.113	5.5674	0.842	0.286	3.4965	0.935
Tear size (ref: < 3 cm)	− 0.884	0.6555	0.178	− 0.031	0.054	0.564	− 7.763	7.5213	0.302	3.155	4.2251	0.455
Suture anchor numbers	0.034	0.3187	0.915	0.004	0.0242	0.853	2.331	3.6778	0.526	− **4.361**	**2.0089**	**0.03**
Time (ref: baseline)												
3 months	− 4.307	0.4283	< 0.001	0.172	0.0387	< 0.001	− 38.436	4.1158	< 0.001	24.403	2.4909	< 0.001
6 months	− 4.150	0.3304	< 0.001	0.162	0.0275	< 0.001	− 30.692	3.2306	< 0.001	15.278	2.2219	< 0.001

Bold values indicate *p *< 0.05

*BMI* body mass index; *CMS* Constant–Murley score; *CCI* Charlson comorbidity index; *EQ5D* EuroQol-5 Dimension; *QDASH* Quick Disabilities of the Arm, Shoulder, and Hand; *Ref* reference; *SSC* subscapularis tendon; *SSP* supraspinatus tendon; *VAS* visual analog scale

#### VAS and EQ5D scores

Table 3 presents the correlations of all baseline parameters with VAS and EQ5D scores. None of the analyzed parameters exhibited significant correlations with postoperative VAS and EQ5D scores within six months postoperatively.

#### QDASH scores

Only BMI had a significant correlation with QDASH scores within six months postoperatively. Increased BMI was significantly correlated with increased QDASH scores (β = 1.561, *p* = 0.03; Table 3).

#### CMSs

Baseline handgrip strength and total suture anchor numbers were significantly correlated with CMSs within six months postoperatively (Table 3). Increased baseline handgrip strength was correlated with increased CMSs, whereas an increased suture anchor number was correlated with decreased CMSs (β = 0.470, *p* = 0.022 and β =  − 4.361, *p* = 0.03, respectively; Table 3). BMI had a negative correlation with postoperative CMSs, although this correlation was nonsignificant (β =  − 0.699,* p* = 0.085; Table 3).

## Discussion

The present study provides several findings: (1) Increased baseline handgrip strength in patients with RCT was correlated with increased CMSs within 6 months postoperatively. (2) An increased total suture anchor number used in ARCR was correlated with decreased postoperative CMSs in the 6-month follow-up. (3) Increased BMI was significantly correlated with increased QDASH scores and negatively correlated with CMSs. Our findings suggest the predictive role of these three parameters for early postoperative shoulder function; higher baseline grip strength predicts more favorable functional recovery after ARCR, whereas a higher total suture anchor number and BMI may predict less favorable early postoperative shoulder function.

Numerous studies have reported a positive correlation between grip strength and rotator cuff function [24–27]. Horsley et al. recruited 27 physically active volunteers with no history of shoulder or upper limb injury and evaluated the relationship between grip strength and lateral rotator strength; a strong correlation was discovered between grip strength and lateral rotator strength at all shoulder positions [25]. Mandalidis and O’Brien observed significantly positive relationships between hand grip isometric strength and isokinetic moments of the shoulder external rotators and abductors, regardless of hand dominance [26]. In a cross-sectional analysis, Manske et al. prospectively enrolled 47 patients diagnosed as having RCT and investigated the relationship between grip strength and several measures of shoulder function [27]. According to their findings, grip strength of the affected side was significantly and positively correlated with the strength of ipsilateral shoulder external rotation and abduction, although no significant correlations were observed between grip strength and patient-reported functional scores [27].

Gripping is a motor task that involves the coactivation of upper extremity muscles, including the muscles of the shoulder, wrist, and hand [26, 34]; a muscle acting at a distal joint can be performed more efficiently when the proximal joints are well stabilized by the surrounding musculature [26]. This mechanism can explain the positive correlation between grip strength and rotator cuff strength. One study supported such a correlation by demonstrating increased grip strength along with increased shoulder stability due to direct strengthening program [35]. As mentioned, grip strength can reflect an individual’s rotator cuff strength; furthermore, preoperative rotator cuff strength and function are prognostic factors for postoperative function in patients undergoing ARCR [16]. Taken together, this may explain the predictive role of baseline handgrip strength in postoperative shoulder functional outcomes, as demonstrated in the present study.

Our analysis revealed that higher grip strength was significantly associated only with higher CMSs; no significant correlation was observed with QDASH scores. CMSs are used to assess four aspects of shoulder function related to shoulder pathology and can be divided into objective (65%) and subjective (35%) domains [33]. Compared with QDASH scores, which are patient self-reported and not shoulder-specific [32], CMSs may reflect shoulder function more precisely and objectively. Furthermore, CMSs include the assessment of shoulder strength [33]. This may explain the significant correlation of grip strength with post-ARCR CMS and the nonsignificant correlation observed with QDASH scores in the present study. Such finding partially aligns with the results of Manske et al., which demonstrated significant correlations between grip strength and the strength of shoulder abduction and external rotation in patients with RCT [27]. However, considering the clinical importance of patient-reported outcomes in surgical patients [36], further investigations into the correlation between grip strength and patient-reported outcome measures of post-ARCR shoulder function is warranted.

In our study, increased BMI is predictive of worse patient-reported functional scores (QDASH) within six months postoperatively; also, increased BMI had a negative correlated trend with objective shoulder-specific functional scores (CMSs). Whether increased BMI is correlated with less favorable shoulder function following ARCR remains controversial on the basis of the current evidence. In a recent retrospective study including 146 patients undergoing arthroscopic or open repair of RCT, Ateschrang et al. found that patients with obesity (BMI > 30) had significantly worse shoulder function and higher re-tear rates than those without obesity with a mean follow-up of 43 months [37]. Within a shorter mean follow-up duration of 16 months, Warrender et al. reported similar results [38]. By contrast, in a study of 213 patients undergoing ARCR, Kessler et al. observed that both obesity (categorical variable, defined as BMI ≥ 30) and increased BMI (continuous variable) did not predict worse functional outcomes at three years postoperatively [39].

Increased BMI is associated with chronic and low-grade systemic inflammation [40]; the chronic inflammation status may interfere with tendon healing and thereby functional recovery following ARCR. Animal studies using rat models have reported poorer tendon and enthesis healing after intentional tendon injury or detachment in obese rats [41, 42]. The negative correlation between BMI and early post-ARCR shoulder function observed in our study may be attributable to impaired tendon healing associated with the chronic subclinical inflammation; in other words, patients with higher BMI may be prone to slower and/or incomplete functional recovery following ARCR. This hypothesis is partially supported by the results of Berglund et al. that patients with obesity had consistently poorer shoulder function after ARCR during the one-year follow-up, although these results achieved statistical significance only at the final assessment [12]. In addition, higher mechanical load on shoulder joints in patients with higher BMI may also affect their functional recovery, considering that these patients generally require more effort and strength to lift or move their arm, which can lead to more stress on the tendon and thus affect tendon healing.

In our study, the overall number of suture anchors utilized for tendon repair was also a predictor of less favorable shoulder function. Generally, the number of suture anchors corresponds to the tear size and the number of torn tendons; if a patient has an RCT with a larger tear size or multiple-tendon involvement, more suture anchors are required for the repair. The total suture anchor number may be more objective than the observed tear size because of the difficulty of measuring the actual tear size through direct arthroscopic visualization during operations. Larger tears and multiple-tendon involvement are predictors of less favorable post-ARCR functional outcomes [16]; our findings regarding the prognostic role of the suture anchor number are consistent with this concept, and further demonstrate a slower functional recovery in the early postoperative period potentially attributable to these factors. However, neither tear size nor the involvement of subscapularis tendon tears (both categorical variables) predicted poorer shoulder function in our study. The nonsignificant results may be attributable to the small and relatively homogenous study population; furthermore, the way of grouping for analyses (such as 1–3-cm vs. > 3-cm tears) may not accurately represent the actual distribution in our study population, which may have also confounded the results.

The strengths of the present study include prospective patient enrollment and outcome assessment. The use of GEE analysis for repeated outcome measurement provides more robustness and is valid when handling missing data. We limited enrollees to those with repairable, full-thickness RCT, and all operations were performed by the same experienced shoulder specialist with expertise in ARCR, which resulted in a relatively homogenous study population. To the best of our knowledge, this is the first prognostic study investigating and reporting a positive correlation between preoperative handgrip strength and postoperative shoulder function in patients undergoing ARCR. Our findings contribute to the knowledge of the correlation between grip strength and rotator cuff function and highlight the potential clinical application of grip strength in patients with RCT. Given its simplicity and objectiveness, the grip strength test may be used in preoperative evaluations to predict postoperative functional recovery.

Some limitations of our study must be addressed. First, the sample size was small, and more than 20% of the patients were lost to follow-up after six months postoperatively, which may have confounded our final results. Second, we only demonstrated the predictive role of baseline grip strength and other parameters in early postoperative shoulder function; whether preoperative grip strength is correlated with long-term or overall shoulder functional outcomes after ARCR remains unknown. Third, we did not investigate the cutoff value of grip strength for the prediction of postoperative outcomes, primarily because of the small sample size.

## Conclusion

This study is the first to demonstrate that high baseline handgrip strength is positively correlated with postoperative functional outcomes in patients undergoing ARCR. Large-scale and well-designed studies are warranted to further investigate the prognostic role of handgrip strength in long-term shoulder function and its potential applications in clinical orthopedic scenarios.

## Data Availability

All data generated or analyzed during this study are included in this published article.

## Abbreviations

RCT: Rotator cuff tear
ARCR: Arthroscopic rotator cuff repair
BMI: Body mass index
CCI: Charlson comorbidity index
VAS: Visual analog scale
EQ-5D: EuroQol-5D questionnaire
QDASH: Quick Disabilities of the Arm, Shoulder, and Hand questionnaire
CMS: Constant–Murley score
SD: Standard deviation
GEE: Generalized estimating equation
