# Correction: Preoperative handgrip strength can predict early postoperative shoulder function in patients undergoing arthroscopic rotator cuf repair

**DOI:** 10.1186/s13018-024-04791-z

**Published:** 2024-06-05

**Authors:** Yu-Cheng Liu, Shu-Wei Huang, Christopher R. Adams, Chung-Ying Lin, Yu-Pin Chen, Yi-Jie Kuo, Tai-Yuan Chuang

**Affiliations:** 1 Department of General Medicine, Shuang Ho Hospital, Taipei Medical University, New Taipei City, Taiwan; 2 Department of Applied Science, National Taitung University, Taitung City, Taitung County Taiwan; 3 Arthrex, Inc., Naples, FL USA; 4 Orthopaedic Department, Naples Community Hospital, Naples, FL USA; 5 Institute of Allied Health Sciences, College of Medicine, National Cheng Kung University, Tainan, Taiwan; 6 Department of Occupational Therapy, College of Medicine, National Cheng Kung University, Tainan, Taiwan; 7 Department of Public Health, College of Medicine, National Cheng Kung University Hospital, National Cheng Kung University, Tainan, Taiwan; 8 Department of Orthopedics, Taipei Municipal Wan Fang Hospital, Taipei Medical University, Taipei, Taiwan; 9 Department of Orthopedics, School of Medicine, College of Medicine, Taipei Medical University, Taipei, Taiwan

**Correction: Journal of Orthopaedic Surgery and Research (2024)19 :270**

10.1186/s13018-024-04750-8.

In this article the statement in the Funding information section was incorrectly given as ' The authors are grateful to Wan Fang Hospital (Grant Numbers: 97TMUWFH-16) for financially supporting this research’ and should have read ‘The authors are grateful to Wan Fang Hospital and Taipei Medical University (Grant number: 113-wf-eva-37) for financially supporting this research’.

The original article has been corrected.

